# Love Stinks: The Association between Body Odors and Romantic Relationship Commitment

**DOI:** 10.3390/brainsci11111522

**Published:** 2021-11-17

**Authors:** Madeleine Keaveny, Mehmet Kibris Mahmut

**Affiliations:** 1 The Matilda Centre for Research in Mental Health and Substance Use, The University of Sydney, Sydney 2006, Australia; madeleine.keaveny@sydney.edu.au; 2 Food, Flavour and Fragrance Lab, School of Psychological Sciences, Macquarie University, Sydney 2109, Australia

**Keywords:** romantic relationships, olfaction, relationship breakdown, Sniffin’ sticks, body odor

## Abstract

Anecdotal reports indicate that women dislike their partner’s body odor (BO) during the breakdown of a relationship; however, whether disliking a partner’s BO is associated with intentions to break up has not been empirically tested. Therefore, the aim of the current study was to investigate, for the first time, whether disliking one’s partner’s BOs is associated with experiencing lower commitment to a romantic relationship. Eighty participants (48 partnered, 32 single and previously partnered) completed self-report questionnaires about their current or previous romantic relationship and the amount of exposure to—and hedonic ratings of—their current or former partner’s BOs. Olfactory function was also tested, and participants smelled and rated various pieces of clothing imbued with a stranger’s BO. The results demonstrated that for participants who had experienced a breakup, historically higher levels of relationship commitment were associated with higher hedonic ratings of a previous partner’s BOs, regardless of the type of BOs. For participants currently in a relationship, lower relationship commitment was associated with higher breakup intentions in response to smelling their partner’s BOs. These preliminary results contribute evidence for the positive association between exposure to a partner’s BOs and favorable hedonic appraisals of BOs; however, further research needs to be conducted in this area to investigate nuances. Lower levels of exposure to one’s partner’s BOs may be more indicative of relationship commitment than exposure to hedonically unpleasant BOs of one’s partner. The findings are discussed with reference to their implications for interventions in relationship breakdown.

## 1. Introduction

Women rate liking someone’s body odor (BO) as the most important physical factor driving sexual attraction and mate choice, while men report that smell is as equally important as physical appearance, demonstrating the influence of BOs in the early stages of a romantic relationship [1,2]. In established relationships, humans may utilize chemosignal cues from their partner’s BO to detect emotional states and subsequently respond effectively, guiding relationship-maintaining behaviors [3,4]. However, research is lacking in the area of relationship breakdown [5], which refers to the dissolution of a romantic relationship. As over 85% of adults have experienced a breakup of a romantic relationship and suffered negative psychological effects as a result, it is of great importance to examine the processes that may be involved in relationship breakdown in order to inform relationship counselling and breakup intervention strategies [6]. While anecdotal accounts suggest that women find their partner’s BO disgusting during the breakdown of their relationship [7], to date, no empirical studies have directly examined this association. As such, the principle aim of this study was to determine whether the hedonic appraisal of a romantic partner’s BOs is associated with the level of commitment to the relationship. Specifically, we predict that lower levels of liking a partner’s BOs will be associated with lower relationship commitment.

According to evolutionary theory, selecting an appropriate and high-quality mate is essential for reproductive success [8]. Consistent with this theory, researchers found that individuals who perceived their partner as having high mate value were more committed to their relationship, more willing to forgive, and had higher relationship satisfaction than those who perceived their partner as having low mate value [9]. Therefore, the selection of an appropriate and high-quality mate may reduce the chances of breakdown. Additionally, if perceptions of a partner’s mate value are altered over the course of the relationship, this could lead to a reassessment of the relationship and trigger relationship breakdown [9].

When selecting a mate, physical health is a major consideration in assessing the suitability of a potential partner [8]. A physically healthy mate is more likely to produce healthy offspring and have the capacity to aid in rearing and providing for offspring [8]. Accordingly, human BOs act as chemosensory cues that signal disease and may subsequently alter patterns of interpersonal interaction [10,11]. In a study by Olsson and colleagues [11], participants rated the BO of donors injected with a placebo as healthier than the BO of donors injected with endotoxin that activated the innate immune response that occurs in sick people, resulting in foul-smelling BO. Thus, people associate foul-smelling BOs with poor physical health, and it follows that this may decrease the individual’s perceived mate value in the eyes of a potential partner [11]. If an individual in an established relationship develops a physical condition that causes them to produce foul-smelling BOs (e.g., typhoid fever), this could lead to avoidance of them as a perceived contaminated source by their partner [11]. Indeed, poor health is a commonly cited reason for divorce of married couples [12].

Body odors relay information about a potential partner’s diet as well as physical health, with the BO of individuals on a vegetarian diet being rated as more attractive than those on a carnivorous diet [11,13]. Therefore, if a partner changes their diet or their health is compromised during a relationship and they develop an unpleasant BO as a result, the other individual may subsequently find their partner less sexually appealing and perceive them as having lower mate value. This could lead to avoidance of the partner and ultimately, if the issue is not resolved, the breakdown of the relationship.

Relationship breakdown has been shown to have a substantial negative impact on psychological, physical, and emotional health [14]. There are various reasons for romantic relationship breakups with the most commonly cited reasons for divorce being infidelity, domestic violence, excessive conflict, poor health, and lack of commitment [12]. Similarly, a meta-analysis found that relational factors were the most robust predictors of relationship breakdown, with commitment being a notable determinant [14]. This is in line with Rusbult’s Investment Model (IM) of romantic relationship commitment and stability [15].

The IM is conceptually based in interdependence theory, which posits that relational exchanges are based on assessments of cost and benefit and that these assessments can influence decisions pertaining to relationship outcome [16,17]. Relationship satisfaction, quality of alternatives, and investments may influence the level of commitment in a relationship [18,19]. Commitment can be understood in the context of a romantic relationship to mean the intent to remain in or to leave the relationship [15,19]. Strong commitment can positively affect a relationship by influencing the responses of partners to relationship conflict, with greater commitment encouraging forgiveness in a relationship, and thus, decreasing the chances of relationship breakdown [18,19]. The IM has been established to be reliably predictive of relationship outcomes, as measured by commitment processes, in longitudinal studies up to fifteen years duration [20,21,22,23]. Additionally, a meta-analysis established the robustness of the IM as a predictor of relationship dissolution across populations and relationship type, with a substantial amount of variance accounted for by commitment level (meta-analytic *r* = 0.47) [19]. The IM posits commitment as the primary driver of maintenance behaviors within a relationship, leading to relationship persistence versus breakdown [24].

The role of BOs in romantic relationships may be most crucial in the sexual interactions of a couple as this is where exposure to one another’s BOs is greatest due to increased physical contact and intimacy [25]. A longitudinal study found that sexual satisfaction is positively associated with relationship satisfaction and commitment in couples [26]. Sexual satisfaction was associated with a decreased likelihood of relationship breakdown although this association was only significant for men, possibly due to men placing greater importance on quality of sexual interactions [26]. Furthermore, frequency of sexual interactions was found to predict relationship stability and persistence [26].

Quality sexual interactions enhance levels of commitment and reduce the chances of relationship breakdown [17,20,26]. Increased olfactory sensitivity was linked to heightened pleasure during sex in both men and women and heightened orgasm frequency in women [25]. This displays how olfaction plays a key role in enjoyment of pleasurable experiences such as sexual intercourse, highlighting the role of chemosensory exchanges in romantic relationships in which sexual activity is applicable. Thus, if an individual dislikes the BO/s of their partner, the frequency and pleasure of sexual interactions may decline, thereby increasing the likelihood of relationship breakdown [1].

People report being disgusted at the idea of having sexual relations with a partner who exhibits symptoms of disease [27]. Physical symptoms that indicate the presence of a sexually transmitted disease, including BOs, can reduce the perceived mate value of a partner and damage the intimate relations of a couple, potentially spurring relationship breakdown [9,10]. Symptoms of disease that produce odor may be especially effective at evoking disgust as, unlike the other senses, olfactory evoked disgust does not habituate [28]. Body odors from dirty armpits, clothes, and feet can evoke disgust and are associated with poor personal hygiene in Western culture [10,29]. While personal standards of hygiene may be socially learned and dependent on culture, the elicitation of disgust in association with feelings of dirtiness may lead to disease-avoidance behaviors [10,29].

In accordance with the disease-avoidance model of disgust, detection of BOs that are perceived as disgusting can lead to avoidance of the source of the odor [10]. Avoidance behaviors act to minimize the risk of transmission of disease [30]. Research by Andrews and colleagues suggests that there is an inhibitory effect of disgust on sexual arousal [31]. Experiencing disgust as a response to perceived pathogenic stimuli may motivate escape and avoidance behaviors as well as divert attention from—and prevent the processing of—sexually arousing stimuli [31]. This avoidance and the associated negative feelings could potentially lead to reduced levels of frequency and quality of both emotional and physical intimacy in a romantic relationship [11]. Subsequently, this may act as a relationship stressor and potentially precipitate relationship breakdown.

Romantic intimacy avoidance due to disliking a partner’s BO has had minimal focus in past research. When considering sexual activity in a previous study [1], females rated BO as the most able to negatively affect sexual desire compared to all other sensory experiences, highlighting the importance of odors in sexual interactions. Similarly, another study found that the experience of pleasant touch decreased in the presence of a foul-smelling odor [32]. The pertinence of olfactory cues in pathogenic avoidance was highlighted in a study that found participants indicated greater proclivity to use condoms when exposed to foul-smelling odor, above and beyond their preconceived views on condom use [33]. In sum, these findings highlight the role of BOs in sexual interactions and how an individual with unpleasant BOs may experience avoidance by their partner, particularly if the partner is female.

The role of olfaction in relationships can be understood by studying individuals without a sense of smell (anosmics). Isolated congenital anosmia (ICA) is characterized by an absence of olfactory percept due to an aplastic or hypoplasic olfactory bulb [34]. In comparison to individuals with normal olfactory ability, male anosmics reported a significantly reduced number of sexual partners, which may be due to their reported enhanced social insecurity as well as their increased prevalence of reported depression [28,35]. Moreover, female anosmics report greater relationship insecurity than non-anosmic females [28]. Poorer olfactory ability may be linked to a heightened risk of relationship breakdown as relationship insecurity is a predictor of relationship breakdown [35]. Studies with individuals who have an impaired sense of smell (hyposmics) also offer valuable insight into the role of olfaction in romantic relationships [36]. Hyposmic individuals report a reduced sexual desire; however, this may be linked to the elevated depression that they also report [37]. Interestingly, individuals with superior olfactory ability have been found to rate sexual experience as more pleasant than those with impaired olfactory ability [25]. Therefore, smell may play a key role in maintaining intimate relationships by providing feelings of closeness and security.

Overall, the literature suggests that BOs play a key, albeit subtle, role in romantic relationships by guiding the initiation, maintenance, and breakdown of partnerships. BOs can influence mate selection through olfactory cues of compatibility and mate value, enhancing the likelihood of quality and long-term relationships. Additionally, BOs continue to play an ongoing role in the maintenance of romantic relationships by guiding interpersonal interaction in couples, potentially influencing the outcome of the relationship.

### Overview of Current Study

The principle aim of this study was to investigate whether greater dislike of a partner’s BOs is associated with lower commitment to the relationship. To examine this association, participants completed questionnaires on their relationship characteristics and commitment, as well as their perception of—and exposure to—their partner’s various BOs. Participants’ olfactory ability was also measured to control for the influence of perception on ratings of partner BOs. The study included partnered and single participants who had previously been in a relationship to ensure people who had experienced a relationship breakdown were included in our study. In line with past research, it was hypothesized that individuals with greater dislike and exposure to their partner’s unpleasant BOs would report lower commitment to the relationship.

## 2. Method

### 2.1. Participants

Eighty undergraduate psychology students from Macquarie University participated in the study for course credit. The total sample consisted of 41 females and 39 males aged between 18 and 58 (*M* = 21.9, *SD* = 6.93). Forty-eight participants were currently in a romantic relationship (30 females; M_age_ = 22.4, SD_age_ = 7.94), and 32 participants were currently single, but had previously been in a romantic relationship (11 females; M_age_ = 21.0, SD_age_ = 5.5). Seventy-two participants were heterosexual, four identified as homosexual and four identified as bisexual. Macquarie University Ethics Committee approved this study, and all participants gave written informed consent.

#### Participant Recruitment

Only individuals who were currently in, or who had previously been in, a romantic relationship were invited to participate given experience in relationships was a requirement for this study. The sample was obtained through two recruitment methods. Initially, first year psychology students voluntarily signed up via an online recruitment system and 64 participants were recruited using this method. The second recruitment method involved inviting first year psychology students who had completed a screener survey that included questions regarding current or previous romantic relationships. A total of 16 participants were recruited from the screener survey.

### 2.2. Materials and Measures

#### 2.2.1. Demographics and Medical History

Participants completed a three-item survey asking for gender, sexual orientation, and relationship status. A medical history interview was used to obtain data on any current or previous conditions that may impact olfactory function (e.g., nasal allergies, nasal surgery, or smoking status).

#### 2.2.2. Sniffin’ Sticks

Olfactory ability was measured using the “Sniffin’ Sticks”, which is a standardized instrument that has shown good validity and test–retest reliability with extensive normative data [38]. Participants completed Sniffin’ Sticks’ three tests: odor thresholds (acuity), odor discrimination, and odor identification. Felt-tip pens that contained odorants were presented approximately 2 cm below the nostrils for approximately 3 s. Scores could range from 0 to 16 on each of the three tests, with higher scores indicating better performance. A total olfactory ability score was formed, which was the sum of all three tests (termed TDI).

#### 2.2.3. Relationship Characteristics Survey (RCS)

An eight-item questionnaire was developed to obtain relationship characteristic information about a participant’s current partner or, if they were single, their most recent, previous partner. The questions asked are listed below with the response options in brackets: (1) relationship duration (0–6 months; 6–12 months; 1–2 years; 2–3 years; 3–4 years; 5+ years); (2) physical (living) proximity to partner (living together; < 15 min drive; 15–30 min drive; 30–60 min drive; 1–2 h drive; 2+ hours’ drive); (3) frequency of contact (daily; 2–3 times per week; once a week; a few times a month; once a month; < once a month); (4) whether the couple were sexually active (yes; no); (5) the number of times the participant cheated on their partner (0; 1; 2; 3; 4+); (6) the number of times the participant’s partner cheated on them (0; 1; 2; 3; 4+); (7) the number of times their relationship ended (0; 1; 2; 3; 4+); and (8) frequency of comfort smelling, that is, intentionally smell clothing/bedding items that their partner’s smell is imbued with (0 = never; 3 = sometimes; 7 = daily). Single participants were asked an additional question—how long ago their previous relationship ended (0–6 months; 6–12 months; 1–2 years; 2–3 years; 3–4 years; 5+ years). Note that the wording of questions was adapted based on whether a participant was currently partnered or single but previously in a romantic relationship.

#### 2.2.4. Investment Model Scale (IMS)

To assess relationship commitment, participants completed a shortened version of the Investment Model Scale (IMS) [18]. The IMS contains 22 items and four subscales: the Relationship Satisfaction subscale had five items (e.g., “My relationship is close to ideal”); the Quality of Alternatives subscale had five items (e.g., “The people other than my partner with whom I might become involved are very appealing”); the Relationship Investment subscale had five items (e.g., “I feel very involved in our relationship—like I have put a great deal into it”); and the Relationship Commitment subscale had seven items (e.g., “I would not feel very upset if our relationship were to end in the near future”). Note that while the overall IMS score is a strong predictor or relationship breakup, the Relationship Commitment subscale alone is equally as predictive of relationship breakup [18].

Participants rated their level of agreement for each statement on a nine-point scale ranging from to do not agree at all (0) to agree completely (8). Note that for currently single, but previously partnered participants, the items were worded in the past tense. After reverse-scoring items, the scores for each subscale were computed by summing the response options on the relevant items. A total score was also calculated by summing the four subscale scores (termed IMS Total). The subscales scores could theoretically range from 0 to 40, except for the Relationship Commitment subscale, which could range from 0 to 56. The IMS Total score could theoretically range from 0 to 176. Higher scores indicate greater overall relationship commitment. The IMS Total score demonstrated good internal consistency for both partnered participants (*a* = 0.89) and single participants (*a* = 0.84).

#### 2.2.5. Object Ratings Task (ORT)

This measure was included to record a behavioral response to actual objects in addition to the collected self-report data on BO exposure. Specifically, the task was designed to measure participants’ liking of and disgust towards three sports-sweat-imbued objects (i.e., sneakers, cotton socks, and a cotton T-shirt) they would likely be exposed to from their partner/previous partner. We did not ask participants to bring in these items of clothing to ensure the standardization of stimuli and allow for meaningful comparisons between participants. The findings of our previous studies, in which participants rated numerous T-shirts imbued with the BO (underarm, exercise sweat) of different strangers [39,40], indicated that this task would be a valid measure of approach (based on hedonic “like” ratings) and avoidance (based on disgust ratings) of clothing items that may smell unpleasant. This measure was important because it may help clarify whether a generalized disgust of potentially unpleasant body odors is reflected in the real-life ratings of partner’s BOs.

To imbue the three items with BO, the male Experimenter (MKM) wore all three objects (i.e., both left and right sneakers and socks, plus T-shirt) simultaneously while engaging in aerobic and strength-conditioning exercises for approximately 60 min. These exercises resulted in significant dampening from sweat of all three object types.

The items (i.e., sock, shoe, and T-shirt) were presented to participants by the Experimenter (MK), one at a time, and they were asked to make three ratings based on the following questions: (1) “Visually, how much do you like the object?” (2) “If you had to pick up the object to move it, how disgusting would you find this task?” (3) “How sexy do you find the smell of the object?” The response options for each question were on a seven-point scale ranging from not at all (0) to very much (6). Three variables were created from this measure (i.e., Visually Like, Touch Disgust, and Sexy Smell) by summing the response option scores across each of the three objects to produce a total score. For example, for the Visually Like variable, if a participant gave a rating of “very much” (i.e., a score of 6) for each of the three objects (i.e., sock, shoe, and T-shirt), their score on this variable would be 18 (i.e., 6 × 3). We collapsed across the three objects because of the high inter-correlations among ratings. Therefore, scores for each of the five variables could range from 0 to 18 where higher scores reflect stronger endorsement of the question.

All objects were always stored in zip-lock plastic bags and only opened for the presentation of objects, which was undertaken in a random order. When the objects were not being used, they were stored in a refrigerator (at approximately 4 degrees Celsius) and were removed from refrigeration at least 30 min prior to use.

#### 2.2.6. Experiences of Partner’s Body Odors Survey (EXBO)

A survey was designed to assess participant’s frequency of exposure to eight of their partner’s BOs that are often perceived as unpleasant based on previous research including sports sweat, breath, urine, feces, flatulence, feet, socks, and general body odor [41]. Participants were asked four questions: (1) “How often have you been exposed to this body odor?” (2) “How much do you like this body odor?” (3) “When smelling this body odor, to what extent did it make you feel like breaking up with them?” (4) “How sexy do you find this body odor?” The response options were presented on a seven-point scale for each question. For the frequency of BO exposure questions, the response options ranged from not at all (0) to daily (6); for the BO liking and BO sexy questions, the response options ranged from not at all (0) to very (6); and for the breaking up questions, the response options ranged from not at all (0) to very much (6). For currently single participants, the items were worded in the past tense. Four variables were created from this measure (i.e., BO Exposure Frequency, BO Exposure Liking, BO Exposure Breakup, and BO Exposure Sexy) by summing the response option scores across each of the eight BOs to produce a total score. For example, for the BO Exposure Frequency variable, if a participant reported being exposed to each of the eight BOs daily, their score on this variable would be 48 (i.e., 8 × 6). Therefore, scores for each of the four variables could range from 0 to 48, where higher scores reflect stronger endorsement of the question. The mean scores for this measure were not calculated because the scores were not meaningful given that not all participants were exposed to each of the eight BOs listed.

### 2.3. Procedure

This study was run over a one-hour session in a Macquarie University lab. Participants completed all self-report measures on a laptop using Qualtrics software. The study measures were completed in the following order: demographics survey, medical history interview, odor threshold test, IMS, odor discrimination test, ORT, EXBO, and finally, the odor identification test. Note that the order of presentation of the ORT and EXBO was counterbalanced to reduce the influence of order effects.

### 2.4. Data Analysis

The first series of analyses were conducted to determine whether partnered and single participants differed on any of the measures they completed. A one-way analysis of variance (ANOVA) was used to test for differences based on relationship status in Sniffin’ Sticks, IMS, ORT, and EXBO. Chi-squared analyses were used to test for relationship status differences on the RCS given the categorical nature of response options. The distribution of scores for each variable were not normally distributed so non-parametric Spearman’s partial correlations were performed, controlling for the influence of olfactory ability using the TDI score. Response options on the comfort smelling question were clustered around the anchors so a more meaningful representation of the data was constructed by transforming the variable into a categorical variable with three levels (0 = never; 1–3 = rarely; 4–7 = sometimes). We set the alpha at 0.05 and adjusted the family-wise error rate using a Bonferroni–Holm adjustment.

## 3. Results

### 3.1. Descriptive Statistics: Partnered vs. Single Participants

To check for differences between the two participant groupings, a one-way ANOVA was conducted and revealed there were no significant differences between partnered and single participants on any of the Sniffin’ Sticks tests or the four factors of the Experiences of Partner’s Body Odors Survey (EXBO; see Table 1). However, single (previously partnered) participants had significantly lower scores on all factors (subscales and total score) of the Investment Model Scale (IMS) and significantly higher Sexy Smell (ORT) compared to partnered participants, whereas partnered participants had higher Touch Disgust ratings than single participants (see Table 1). To check whether the significant differences found between partnered and single participants were related to gender, we also compared males and female scores on the IMS and ORT. The results of this one-way ANOVA revealed there were no differences between males and females in any of the IMS factors or in the Total score; however, females had significantly higher Touch Disgust scores than males (10.44 vs. 7.97, *F* = 8.30, *p* = 0.005, *d′* = 0.64).

The chi-squared analyses revealed that partnered and single participants demonstrated similar relationship demographics (see Table 2). For example, participants had been with their current or former partner for 12 or more months, most participants had regular contact (2–3 times per week) with their current or former partner, and the majority of participants were sexually active with their current or former partner. For single participants, their relationships ended between approximately 6 and 12 months before completing the study. To check whether the significant differences found between partnered and single participants (see Table 2) were related to gender, we conducted further chi-squared analyses. These results of these analyses showed that males and females had significantly different scores on one variable examined. Specifically, a significantly higher proportion of females reported never breaking up with a partner and resuming their relationship than males (80% vs. 59%; chi-squared = 7.07, *p* = 0.023, *d’* = 0.60).

### 3.2. Was Greater Liking of Partner’S BOs Associated with Relationship Commitment?

Spearman’s partial correlations were conducted to determine if hedonic liking of one’s current or previous partner’s BOs was associated with commitment as measured by the IMS. The correlations were conducted separately for single ((a) in Table 3) and partnered ((b) in Table 3) participants due to the significant group differences reported earlier. Given that variation in olfactory ability may influence the detection of odors in the environment, we statistically controlled for olfactory ability (TDI score) in these partial correlations to remove any influence on BO exposure or liking and relationship commitment.

The results for single participants (see ((a) in Table 3) revealed that overall, most of the IMS subscales and the Total score were positively correlated with the subscales of the Exposure to Partner Body Odor survey (EXBO), except for the correlations with the BO Breakup variable. The significant positive correlations found between the IMS Total score and the BO Liking, BO Sexy, and BO Exposure variables indicated that higher degrees of relationship commitment were associated with higher hedonic ratings of partner BOs and lower breakup intentions in response to exposure to unpleasant partner BOs. While a similar pattern of findings was revealed for partnered participants (see ((b) in Table 3), the IMS Total score was not significantly correlated with BO Exposure, BO Liking, or BO Sexy. However, a significant negative correlation was found between the IMS Total score and the BO Breakup variable, indicating that higher ratings of wanting to break up with one’s partner after smelling their unpleasant BOs were associated with lower levels of relationship commitment.

The within-measure correlations were as expected for both single and partnered participants. For example, for the EXBO survey, higher BO Sexy ratings were positively and significantly correlated with BO Liking ratings. Interestingly, significant, positive correlations were also found between BO Exposure and BO Liking (partnered participants only) and BO Sexy (single participants only) ratings, indicating that higher degrees of exposure to BOs were associated with more favorable ratings of BOs (see Table 3).

## 4. Discussion

In line with our hypothesis, higher degrees of relationship commitment were associated with higher levels of exposure to—and hedonic ratings of—a partner’s BOs and lower breakup intentions in response to exposure to a partner’s unpleasant BOs; however, this finding was only applicable for single participants. For those who had experienced a breakup, currently single participants still reported a positive association between exposure to partner’s BOs and more favorable hedonic ratings of their BOs, and relationship commitment. For currently partnered participants, we saw similar patterns albeit non-significant results. Interestingly, we did find that partnered participants reported higher ratings of wanting to break up with one’s partner after smelling their unpleasant BOs, as associated with lower levels of relationship commitment. These results suggest that independent of relationship status as well as relationship characteristics, including relationship length, sexual activity, contact frequency, partner proximity, and the type or unpleasantness of a partner’s BOs, greater exposure to—and favorable hedonic ratings of—a partner’s BOs was associated with greater commitment to the relationship.

While we found no differences between the single and partnered groups in initial analyses regarding exposure to—and liking of—a partner’s BOs, or relationship characteristics, further analyses teased out some interesting results. Unsurprisingly, levels of relationship commitment did differ significantly between single versus partnered participants and there is the obvious possibility that these groups of participants may historically appraise their relationship nuances very differently dependent on their current relationship status. Gender did not appear to influence these significant group differences, with no differences between males and females on any of the IMS factors or Total score. Olfactory ability did not differ between groups and was not significantly associated with gender. However, females had significantly higher Touch Disgust scores than males, reflecting past studies reporting females rating unpleasant BOs as most pertinent in triggering disgust-avoidance during sexual encounters, suggesting females have lower disgust sensitivity than males specifically regarding BOs [1].

Mahmut and colleagues found that women rated their partner’s BO as more pleasant than that of an opposite-sex stranger’s BO [39]. This suggests that olfactory cues can influence relationship persistence by diverting attention from a potential alternative partner. Indeed, a previous study found that when asked to identify BOs, women were better at identifying their boyfriend’s BO than that of an opposite-sex friend [42]. This is in line with the notion that romantic love deflects attention away from alternative mates and that this process extends to the processing of olfactory cues [42]. For the current study, single participants rated a real stranger’s BO as sexy at higher ratings than those made by currently partnered participants, supporting the notion that partnered participants would rate a potential alternative partner’s BO differently due to cued attention diversion, possibly supporting commitment to the relationship.

The significant relationship found between variables of BOs exposure and liking, in terms of relationship commitment, was not found for participants currently in a relationship. Yet, partnered participants reported one finding paralleled in the single sub-sample, namely that there was a significant, negative association between relationship commitment and ratings of wanting to break up after smelling a partner’s unpleasant BOs. While there are no directly comparable studies, our results are consistent with at least one study that found that partnered women liked their current partner’s BO no more than single women liked a platonic friends’ BO [39]. Their findings, in line with ours, indicate that the valence (i.e., pleasantness or unpleasantness) of the body odors subjects are exposed to is not as important as exposure per se [39]. Indeed, a robust finding in olfaction research is that familiarity is strongly associated with pleasantness ratings of an odor [43]. For both groups of participants, greater BO Exposure were associated with more favorable ratings of BOs, with BO Exposure being associated with BO Liking (partnered participants) and BO Sexy (single participants), reinforcing previous research that simply greater exposure to BO can increase hedonic liking [43].

A picture that is beginning to emerge from an extrapolation of the current and previous empirical findings is that psychological dislike of a person precedes and predicts finding their BO unpleasant or even aversive. For example, research has shown that body odor disgust sensitivity was associated with xenophobia [44]. Anecdotal evidence suggesting that psychological factors precede dislike of perceptual experiences originates from MKM receiving information from a person who found their partner’s BO disgusting after learning of their infidelity and during the subsequent divorce proceedings. However, once the divorce proceedings were finalized, the (now divorced) couple engaged in passionate sexual encounters with each other and the adulterer’s BO was no longer perceived as disgusting. Given that single participants reported significantly higher rates of infidelity by their partner, compared to those in the partnered group, it is possible that the significantly lower hedonic ratings of their partner’s BOs were triggered by a psychological dislike due to their infidelity. However, the low numbers of participants that reported infidelity (eight in the Single group and one in the Partnered group) meant that this line of inquiry could not be meaningfully explored further.

Various caveats should be considered when interpreting these findings. First, a larger sample size would have been ideal; however, given the difficulty of this type of research, these relatively smaller samples were deemed sufficient. Second, due to the correlational nature of this study, directionality cannot be assumed. Exposure to, and liking of, a partner’s BOs may influence relationship commitment; however, it may be that relationship commitment influences exposure to, and liking of, a partner’s BOs. While potentially confounding variables were controlled for in this study, such as olfactory ability, it was impossible to account for all potentially confounding variables due to the novel nature of the study. In addition, this study’s measurement of BO exposure may prove a limitation. The EXBO, a novel scale, only asked participants about eight specific BOs their partner emitted. This may have neglected other BOs that participants may have been exposed to as they were asked to recall from memory how often they were exposed to their partner’s BOs. Additionally, participants responded on a relatively arbitrary scale with no reference odor for ratings comparison, possibly reducing the accuracy of responses, especially hedonic ratings. Another novel scale used, the ORT, may have limited validity due to including solely male BO while neglecting the opportunity for ratings of female BO. Considering the gender parity of the sample, and the multitude of olfaction literature evidencing gender differences in perceptions of BO, it would have been ideal to include both male and female BO in the task for participants to rate. Finally, all participants were undergraduate University students. The average relationship length of this sample was only 6–12 months, and the average age of participants was 22 years. This narrow range of age and relationship length may limit the generalizability of the study’s findings. While this age range is a time when people are most likely to search for and meet long-term partners, youth mating strategies may differ from the general population [45].

### 4.1. Implications and Directions for Future Research

The results of the current study could inform therapeutic interventions for romantic relationships, especially for people who have low relationship commitment, an impaired sense of smell, or unpleasant BOs. As relationship breakdown can have detrimental psychological, social, and physical effects for people [14], it is important to understand the factors that may spur relationship breakdown. This study’s finding that even people who have experienced a relationship breakup also reported an association between relationship commitment and greater exposure to—and liking of—their partner’s BOs prior to relationship breakdown, suggesting that while intimacy within a relationship is important for maintaining the bond between partners and reducing the likelihood of relationship breakdown, there are potentially other factors at play. Such factors may include partner infidelity, a relationship characteristic that was reported significantly more within the Single than the Partnered group, although further research is warranted to establish this finding due to our small sample size. As disliking a partner’s BOs can lead to a decrease in frequency of contact, interventions could be made to treat health conditions that may result in unpleasant BOs or to develop perfumes that could mask unpleasant BOs and increase sexual attractiveness [46]. Indeed, that perfumes interact with people’s natural BO to create a unique odor combination, amplifying their natural scent [46]. Future studies could investigate whether greater exposure to—or liking of—a partner’s BOs specifically relates to relationship commitment.

### 4.2. Conclusions

Overall, this study made a unique contribution to the literature on the function of BOs in romantic relationships by demonstrating that regardless of the type or unpleasantness of BOs, greater historic exposure to—and hedonic liking of—a partner’s BOs is related to greater commitment to the relationship for those who have experienced a breakup. For individuals currently in a relationship, lower relationship commitment was associated with higher ratings that the hedonics of a partner’s BOs made them want to break up. Furthermore, greater exposure to a partner’s BOs was found to be associated with more favorable hedonic ratings of BOs, adding to substantial past research evidencing this link. It may be that increased frequency of exposure to one’s partner’s BOs is driven by hedonic liking of these BOs, potentially setting up a type of positive-feedback loop that perhaps serves to increase relationship commitment. Further research should be conducted in this area to tease out the nuances of these preliminary findings. The current study’s findings encourage us to not just stop and smell the roses but to stop and smell our partner.

## Figures and Tables

**Table 1:** Descriptive statistics and comparisons of Sniffin’ Sticks, IMS, ORT, and EXBO scores by relationship status.

Measure	Partnered (*n* = 48) Mean (SD)	Single (*n* = 32) Mean (SD)	F-Value (*d*′)
Sniffin’ Sticks			
Odor Threshold	7.56 (3.00)	7.84 (3.26)	<1 (0.09)
Odor Discrimination	10.50 (2.07)	11.34 (1.70)	3.66 (0.44)
Odor Identification	11.79 (1.49)	12.13 (1.72)	<1 (0.21)
TDI	29.79 (4.39)	31.21 (4.48)	1.97 (0.32)
Investment Model Scale			
Relationship Satisfaction	33.48 (6.43)	22.87 (7.81) **	43.93 (1.51)
Quality of Alternatives	26.67 (8.52)	21.72 (7.93) *	6.83 (0.60)
Relationship Investment	25.63 (7.47)	20.97 (7.24) *	7.64 (0.63)
Relationship Commitment	49.27 (9.87)	37.59 (12.93) **	20.92 (1.04)
Investment Model Scale Total	150.04 (24.31)	118.16 (23.30) **	34.13 (1.33)
Object Rating Task			
Visually Like	8.92 (2.89)	9.72 (2.57)	1.61 (0.29)
Touch Disgust	10.00 (3.99)	8.09 (3.79) *	4.56 (0.49)
Sexy Smell	3.77 (1.21)	4.63 (2.14) *	5.20 (0.52)
EXBO Survey			
BO Exposure Frequency	21.02 (8.99)	19.19 (8.88)	<1 (0.20)
BO Exposure Liking	10.08 (5.39)	10.63 (6.19)	<1 (0.09)
BO Exposure Breakup	0.98 (2.54)	2.25 (3.39)	3.67 (0.44)
BO Exposure Sexy	7.71 (4.92)	9.34 (6.06)	1.76 (0.30)

Note. The F-values were obtained from one-way ANOVA and reflect comparisons of partnered vs. single participants analyses. *d′* = Cohen’s D effect size. TDI = sum of odor thresholds, discrimination, and identification scores. EXBO = Experiences of Partner’s Body Odors. * *p* < 0.05, ** *p* < 0.01.

**Table 2:** Descriptive statistics and comparisons of Relationship Characteristics Survey data by relationship status.

	Partnered (*n* = 48)	Single (*n* = 32)	Chi-Squared (*d′*)
Current/previous partner distance (driving)			
Live(d) with	13%	16%	2.05 (0.28)
Less than 30 min	54%	66%	
More than 30 min	33%	18%	
Current/previous relationship length			
Less than 12 months	44%	50%	<1 (0.12)
More than 12 months	56%	50%	
Current/previous partner contact frequency			
Daily	31%	28%	<1 (0.05)
2–3 times per week	56%	59%	
Once per week or less	13%	13%	
Participant cheated on current/previous partner			
Never	90%	84%	<1 (0.18)
Once or more	10%	16%	
Current/previous partner cheated on participant			
Never	98%	75%	10.10 ** (0.78)
Once or more	2%	25%	
Sex with current/previous partner (Yes)	92%	72%	5.53 * (0.56)
Times broke-up and resumed relationship			
Never	79%	56%	7.58 * (0.63)
Once	15%	16%	
Twice or more	6%	28%	
Comfort Smelling			
Never	25%	25%	6.30 * (0.21)
Rarely	4%	22%	
Sometimes	71%	53%	

Note. *d′* = Cohen’s D effect size. * *p* < 0.05, ** *p* < 0.01.

**Table 3:** (**a**). Spearman’s correlations between BO exposure (EXBO), relationship commitment (IMS), and object ratings (ORT)—single participants (*n* = 32). (**b**). Spearman’s correlations between BO exposure (EXBO), relationship commitment (IMS), and object ratings (ORT)—partnered participants (*n* = 48).

(a)												
Measure	1.	2.	3.	4.	5.	6.	7.	8.	9.	10.	11.	12.
1. BO Exposure	-	0.31	0.56 **	−0.04	0.21	0.18	0.38 *	0.29	0.40 *	0.56 **	0.02	0.39 *
2. BO Liking		-	0.83 **	0.14	0.25	0.24	0.12	0.43 *	0.42 *	0.32	−0.04	0.24
3. BO Sexy			-	0.01	0.35	0.26	0.24	0.45 *	0.50 **	0.39 *	−0.01	0.27
4. BO Breakup				-	−0.20	−0.22	−0.09	−0.22	−0.29	0.08	0.43 *	0.22
5. Relationship Satisfaction					-	−0.06	0.58 **	0.18	0.54 **	0.13	0.04	0.16
6. Quality of Alternatives						-	−0.17	0.42 *	0.45 *	0.15	−0.22	−0.03
7. Relationship Investment							-	0.24	0.55 **	0.21	0.12	0.12
8. Relationship Commitment								-	0.84 **	0.23	−0.05	0.12
9. IMS Total									-	0.32	−0.06	0.26
10. Object Visually Like										-	−0.21	0.49 **
11. Touch Disgust											-	0.05
12. Object Smell Sexy												-
**(b)**												
**Measure**	**1.**	**2.**	**3.**	**4.**	**5.**	**6.**	**7.**	**8.**	**9.**	**10.**	**11.**	**12.**
1. BO Exposure	-	0.42 **	0.23	0.03	−0.15	0.04	−0.04	0.13	0.01	0.14	0.05	0.03
2. BO Liking		-	0.82 **	−0.04	0.16	0.26	0.00	0.24	0.19	0.09	0.07	0.08
3. BO Sexy			-	−0.07	0.10	0.29 *	0.04	0.20	0.19	0.24	0.07	0.29 ^~^
4. BO Breakup				-	−0.28	−0.23	−0.25	−0.28	−0.38 **	0.04	0.05	0.14
5. Relationship Satisfaction					-	0.31 *	0.38 **	0.47 **	0.68 **	−0.22	0.04	−0.10
6. Quality of Alternatives						-	0.22	0.47 **	0.69 **	−0.09	0.02	−0.13
7. Relationship Investment							-	0.49 **	0.67 **	−0.07	−0.02	0.13
8. Relationship Commitment								-	0.84 **	0.01	−0.01	−0.15
9. IMS Total									-	−0.09	−0.00	−0.13
10. Object Visually Like										-	−0.24	0.27
11. Touch Disgust											-	−0.13
12. Object Smell Sexy												-

Note. (**a**) BO = Body odor. * *p* < 0.05, ** *p* < 0.01. Olfactory ability (measured by TDI) was statistically controlled for using partial correlations (except for within-measure correlations). Cells with correlations between two different measures are grey. IMS = Investment Model Scale. The three different bivariate correlations are demarcated with cell borders. (**b**) BO = Body odor. ^~^ *p* = 0.052 * *p* < 0.05, ** *p* < 0.01. Olfactory ability (measured by TDI) was statistically controlled for using partial correlations (except for within-measure correlations). Cells with correlations between two different measures are grey. IMS = Investment Model Scale. The three different bivariate correlations are demarcated with cell borders.

## Data Availability

The data is not available due to confidentiality reasons.
